# A pilot study investigating anterior segment optical coherence tomography angiography as a non-invasive tool in evaluating corneal vascularisation

**DOI:** 10.1038/s41598-020-80099-2

**Published:** 2021-01-13

**Authors:** Hon Shing Ong, Kai Yuan Tey, Mengyuan Ke, Bingyao Tan, Jacqueline Chua, Leopold Schmetterer, Jodhbir S. Mehta, Marcus Ang

**Affiliations:** 1 Singapore Eye Research Institute, Singapore, Singapore; 2 Department of Ophthalmology and Visual Science, Duke-National University of Singapore (NUS), Graduate Medical School, Singapore, Singapore; 3 Singapore National Eye Centre, 11 Third Hospital Avenue, Singapore, 168751 Singapore; 4 Hobart Clinical School, Level 3, 43 Collins Street, Hobart, Australia; 5 School of Chemical and Biomedical Engineering, Nanyang Technological University, Singapore, Singapore; 6 SERI-NTU Advanced Ocular Engineering (STANCE), Singapore, Singapore; 7 Centre for Medical Physics and Biomedical Engineering, Medical University of Vienna, Vienna, Austria; 8 Deaprtment of Clinical Pharmacology, Medical University of Vienna, Vienna, Austria; 9 Institute of Clinical and Experimental Ophthalmology, Basel, Switzerland; 10 School of Material Science and Engineering, Nanyang Technological University, Singapore, Singapore

**Keywords:** Medical research, Translational research, Eye manifestations, Eye diseases, Medical imaging

## Abstract

The current assessment of corneal vascularisation (CV) relies on slit-lamp examination, which may be subjective. Dye-based angiographies, like indocyanine green angiography (ICGA), allows for good visualisation of anterior segment blood vessels. However, ICGA is invasive and can be associated with systemic adverse effects. Anterior segment optical coherence tomography angiography (AS-OCTA) is a non-invasive tool that has been shown to successfully delineate CV. However, there are no previous studies that have reported if AS-OCTA can determine CV stage and activity. We used an established CV model in rabbits to examine serial AS-OCTA scans of CV development and regression following treatment with anti-vascular endothelial growth factor. We compared AS-OCTA derived vascular measurements to that of ICGA determined vessel leakage and CV staging. Our results showed that AS-OCTA vessel densities and vessel branch area significantly correlated with the severity of CV based on ICGA (all p ≤ 0.05). We also found that AS-OCTA vessel densities correlated with ICGA vessel leakage time, following an inverse linear relationship (r^2^ = − 0.726, p < 0.01). Changes in aqueous levels of CXCL-12 and PIGF cytokines significantly correlated with AS-OCTA vessel densities (r^2^ = 0.736 and r^2^ = 0.731 respectively, all p < 0.05). In summary, we found that AS-OCTA derived vessel parameters may be useful for assessing CV severity, while vessel density correlates with CV activity and leakage. Thus, our pilot animal model study suggests that AS-OCTA may be a useful non-invasive imaging tool to provide objective assessment of CV to examine progression or response in treatment, which requires confirmation in clinical studies.

## Introduction

Anterior segment optical coherence tomography (AS-OCT) has become an important imaging technique to assess the cornea and anterior segment of the eye in daily clinical practice^1^. While traditional OCT imaging provides detailed information on the various structures of the eye, recent advancements using de-correlation algorithms of OCT signals has led to its ability to delineate blood vessels as well^1^. The main advantage of OCT angiography (OCTA) is its non-contact and rapid ability to image ocular vasculature without the use of invasive dyes^2^. While OCTA has been used primarily in the imaging of posterior segment vasculature, its use in anterior segment imaging is just emerging^2,3^.

At present, slit lamp photography is the most widely used clinical imaging technique to visualize blood vessels in the anterior segment and ocular surface, such as the cornea, limbus, and conjunctiva^4^. However, as slit lamp photography can only offer two-dimensional images, visualisation of blood vessels can be limited in the presence of corneal or limbal opacities^5–7^. Indocyanine green angiography (ICGA), a dye-based angiography, is another imaging technique that provides good visualisation of blood vessels in the anterior segment^6^. However as ICGA is an invasive technique associated with potential risks to patients^8^, it is rarely used in clinical practice to assess the severity of anterior segment vascularisation^6,9^.

Given the limitations of existing imaging modalities, anterior segment OCTA (AS-OCTA) has been introduced as an alternative rapid and non-invasive imaging tool to objectively image anterior segment vasculature in both healthy and diseased states^1,3,5,9–12^. However, OCTA is unable to detect specific vessel characteristics such as the speed of blood flow or vessel leakage^13^. As such, OCTA has been unable to completely replace dye-based angiographies, since vessel leakage is often used as a surrogate marker of disease severity and activity, especially in the posterior segment^14^. In the anterior segment, studies suggest that OCTA and ICGA can produce comparable quantification of corneal vascularisation with good inter- and intra-observer agreements^15^. Nevertheless, there are currently no studies that have examined the correlation of OCTA vessel parameters with the severity of corneal vascularisation and vessel leakage^16^.

For this study, we hypothesised that AS-OCTA vascular measurements may correlate with corneal vascularisation severity and activity, which would help support the use of AS-OCTA as an alternative to invasive dye-based angiography such as ICGA. Thus in order to test our hypothesis, we conducted serial AS-OCTA measurements of corneal vascularisation development and regression in an established animal model with anti-vascular endothelial growth factor (anti-VEGF) treatment. We then compared these AS-OCTA vascular measurements to corneal vascularisation measurements and leakage based on serial ICGA imaging, and pro-angiogenic aqueous biomarkers.

## Methods

All animals were treated as per guidelines of the Association for Research in Vision and Ophthalmology’s statement for the Use of Animals in Ophthalmic and Vision Research. Experimental protocols were carried out as approved by the SingHealth Institutional Animal Care and Use Committee (IACUC reference number: 2015/SHS/1090) and housed under standard laboratory conditions at the SingHealth Experimental Medical Centre, Singapore General Hospital. This study included 10 eyes of 10 New Zealand white rabbits, Male, 2.0–2.5 kg, 12–15 weeks. All surgeries and imaging evaluations were performed under general anaesthesia with xylazine hydrochloride (5 mg/kg intramuscularly; Troy Laboratories, Smithfield, Australia) and ketamine hydrochloride (50 mg/kg intramuscularly; Parnell Laboratories, Alexandria, Australia). Using a previously established experimental model^5,10,17^, corneal suturing was performed in the right eye of rabbits to induce corneal vascularization.

All rabbits were evaluated every week for 2 weeks for the progression of corneal neovascularization using slit lamp photography (Righton NS-2D slit-lamp camera, Tohoku Right Mfg., Miyagi, Japan), AS-OCTA (Nidek RS-3000, Tokyo, Japan), and ICGA (HRA2, Spectralis scanning laser angiography, Heidelberg Engineering, Heidelberg, Germany) imaging. For AS-OCTA acquisition, the anterior segment lens was used with a previously described scan protocol^18^. Each scan was taken four times in the same quadrant, ensuring a good signal strength. The eye tracking and autofocus functions were deactivated in the acquisition software, and manual adjustments to the Z-motor and focal length positions were made for setting focus onto the cornea. ICGA was captured as a video file for the first two minutes following dye injection^19^. Thereafter, image frames were captured every minute for a duration of ten minutes. Time taken from injection of ICGA to vessel leakage were recorded by two observers (KD and KYT); the average timings of vessel leakage determined by the two observers were subsequently used for analyses.

**Figure 1:**
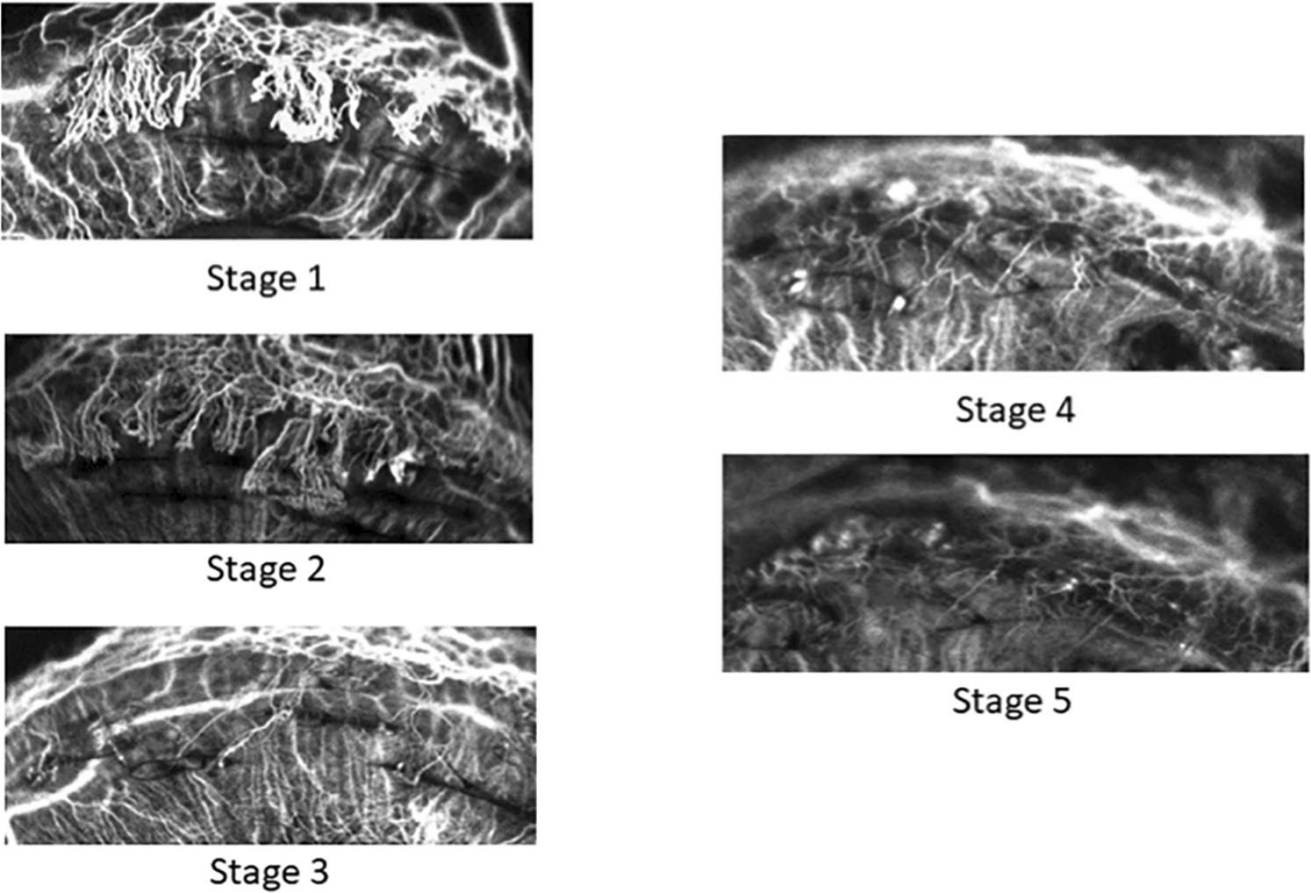
Corneal vascularisation staging determined using indocyanine green angiography^19^. Stage 1: Active young vessels that are newly formed and progressing with a well-defined fine capillary network; signs of leakage and oedema are seen in the surrounding corneal stroma. Stage 2: Active old vessels which appear less bright and reach the lesion but not progressing. Stage 3: Partially regressed vessels with slow blood circulation and are of reduced visibility. Stage 4: Mature vessels that are relatively large with minimal arborisation and regressed or absent capillary networks. Stage 5: Regressed vessels that are very fine white lines mirroring the morphology of the original vessels (ghost vessels).

After development of corneal vascularisation at 2 weeks following corneal suturing, eyes were divided into three groups: a treatment group (aflibercept, n = 3) where eyes received 0.05 ml sub-conjunctival injection of 2 mg aflibercept (Eylea; Bayer Pharma AG; Berlin, Germany); a control group (saline, n = 2) where eyes received 0.05 ml normal saline sub-conjunctival injection; and a non-treatment group (re-suture, n = 5) where eyes had additional corneal sutures to further maintain active corneal vascularisation but received no injections. All eyes were subsequently imaged with slit lamp photography, AS-OCTA, and ICGA for further 2 weeks. The three groups were able to produce various stages of corneal vascularisation staging, based on a previously described guide using ICGA imaging^19^ (Fig. 1):

*Stage 1* Active young vessels that are newly formed and progressing with a well-defined fine capillary network. Signs of leakage and oedema are seen in the surrounding corneal stroma.

*Stage 2* Active old vessels which appear less bright and reach the lesion but not progressing.

*Stage 3* Partially regressed vessels with slow blood circulation and are of reduced visibility.

*Stage 4* Mature vessels that are relatively large with minimal arborisation and regressed or absent capillary networks.

*Stage 5* Regressed vessels that are very fine white lines mirroring the morphology of the original vessels (ghost vessels).

### Aqueous cytokine analysis

Aqueous humour was collected from rabbit eyes under sterile conditions prior to every imaging session. 100 µl of aqueous humour was aspirated using a 30-gauge needle on a 1 ml syringe through an anterior chamber paracentesis. An aliquot of aqueous humour was then stored at − 80 °C within 2 h of collection until further analysis. LUNARIS Human 11-Plex Ophthalmology kit (Ayoxxa Biosystems GmbH, Germany) was used to measure the concentrations of the following known pro-angiogenic and pro-inflammatory human cytokines/chemokines: Ang-2 (Angiopoietin-2), CRP (C-reactive protein), CXCL10 (IP-10, interferon-gamma-induced protein 10), CXCL12 (stromal cell-derived factor 1 alpha), CXCL13 (B lymphocyte chemoattractant), IL-6 (Interleukin-6), IL-8 (Interleukin-8), PlGF (Placenta growth factor), VEGF-A (vascular endothelial growth factor A), CCL2 (MCP-1, monocyte chemotactic protein-1), PDGF-BB (platelet-derived growth factor-BB). The assays were processed and analysed according to the manufacturer’s instructions. 10–20 µl of aqueous humour sample was used in each reaction. Fluorescence intensity (FI) from the immunoassay was acquired and analysed using the kit. Concentrations that were lower than the minimum quantifiable levels were defined as ‘below limits of detection’.

### Image processing

**Figure 2:**
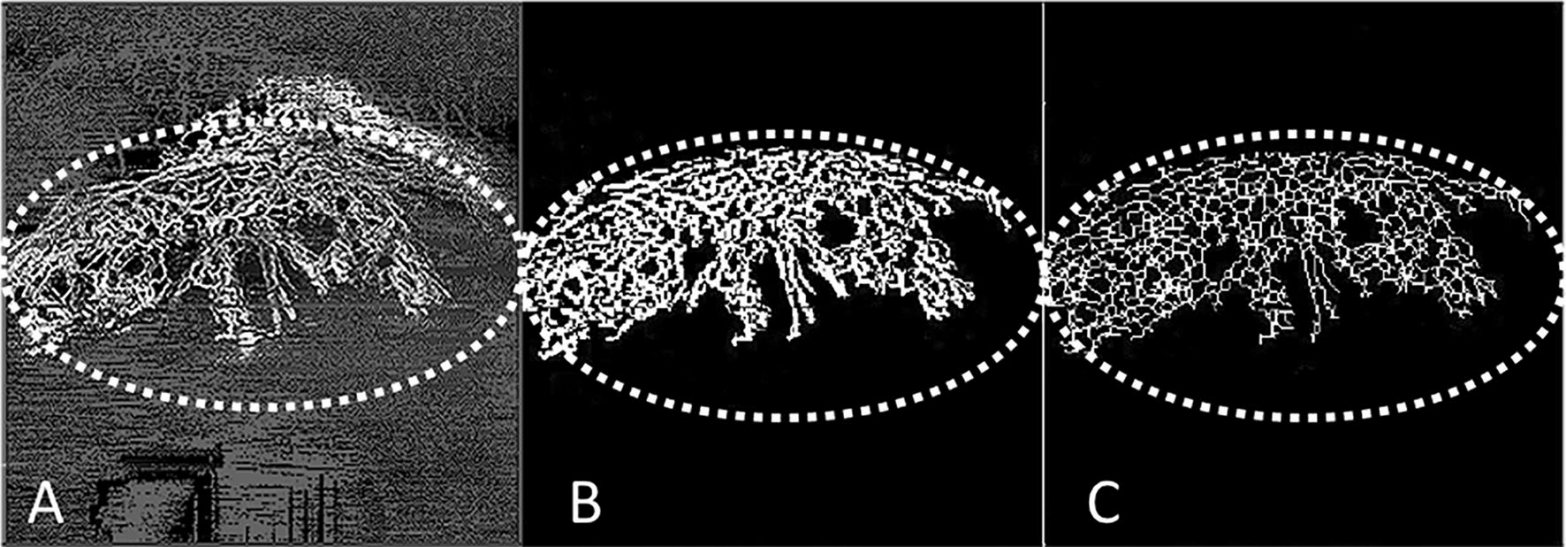
Image processing technique using an in-house automated programme written in MATLAB (Mathworks, Inc., Natick, MA, USA); the region of interest (ROI) is the area demarcated by the dotted line. (**A**) Example of corneal vascularisation captured using anterior segment optical coherence tomography (AS-OCT) angiography (Nidek RS-3000, Tokyo, Japan); (**B**) the overlay image is binarized at the ROI where white pixels represented the blood vessels and black represented the background—vessel density (VD) is the area of white pixels as a percentage of the whole binarized image; (**C**) Skeletonization of the binarized images, where all the vessel segments were reduced to one pixel-wide segment—vessel branch area (VBA) is the total length of the vessel branch as a percentage of the whole binarized image; the VD as a percentage of the VBA, represents the vessel width of the corneal vessels in the ROI.

Image processing and computations were performed in full projection AS-OCTA and ICGA images, using an in-house automated programme written in MATLAB (Mathworks, Inc., Natick, MA, USA) software (Fig. 2). The processing was performed following a similar technique used previously^5,10^. First, speckle noise was removed using a median filter and Gaussian smoothing. Second, top-hat filter was applied to improve signal-to-noise ratio while preserving the image features. Local phase based filter for optimal enhancement of segmented vessels was subsequently applied, and an infinite perimeter active contour model was used to partition the enhanced image into a binary image (vessel pixels, 1; background, 0)^10^. The binarized images over the same overlay region were then cropped and used to compute vessel density, where white pixels represented the blood vessels and black pixels, the background (Fig. 2B). Skeletonization of the binarized images was then performed, where all the vessel segments were reduced to one pixel-wide segment^20^ (Fig. 2C). The sum of skeletonised segments represented the total branching length area of the vessels.

*Vessel density (VD)* was defined as the area of white pixels (corresponding to blood vessels) as a percentage of the whole binarized image [i.e. VD (%) = area of white pixels ÷ area of whole binarized image × 100]^21^ (Fig. 2B). This gives the quantitative value of the overall perfused vessel density in the *en face* image of the corresponding segmented layer. Corneal vessel widths and branch areas were also assessed from the AS-OCTA images^21^. *Vessel branch area (VBA)* was defined as the total length of vessel branches as a percentage of the whole binarized image [i.e. VBA (%) = total length of vessel branches ÷ length of whole binarized image × 100]. This parameter assesses the vessel length of all the vascular branches existent in the segmented *en face* OCT segment. *Vessel width (VW)* was defined as the average vessel calibre width within the *en face* OCT image. It was calculated as the proportion of percentage vessel density over percentage vessel branch area [i.e. VW = VD (%) ÷ VBA (%)]. It represents the approximate vessel diameter of the specific segmented layer.

### Statistical analysis

Linear mixed-effects models and univariate linear simple regression models were performed to model the relationships between AS-OCTA parameters (VD, VBA, and VW) with ICGA-determined stages of corneal vascularisation and the vessel leakage timings, respectively. Data used for these analyses were prior to any subconjunctival injections. Assuming the anticipated effect size between the groups to be two fold, an estimated sample size of four eyes in each group will be needed to give the study 80% power to show a difference between the groups, with a statistical precision of no worse than 0.05. Amongst the two groups, paired sample t-test was performed to compare the changes in mean AS-OCT vascular parameters in the follow-up period. Independent t-test was performed to compare the difference in mean AS-OCT vascular parameters between the treatment and control group following injection. A correlation matrix was constructed using Spearman’s method to show the correlation between the AS-OCTA vascular parameters and aqueous pro-angiogenic cytokine levels, prior to subconjunctival injections. Significance level was set at p < 0.05.

## Results

**Figure 3:**
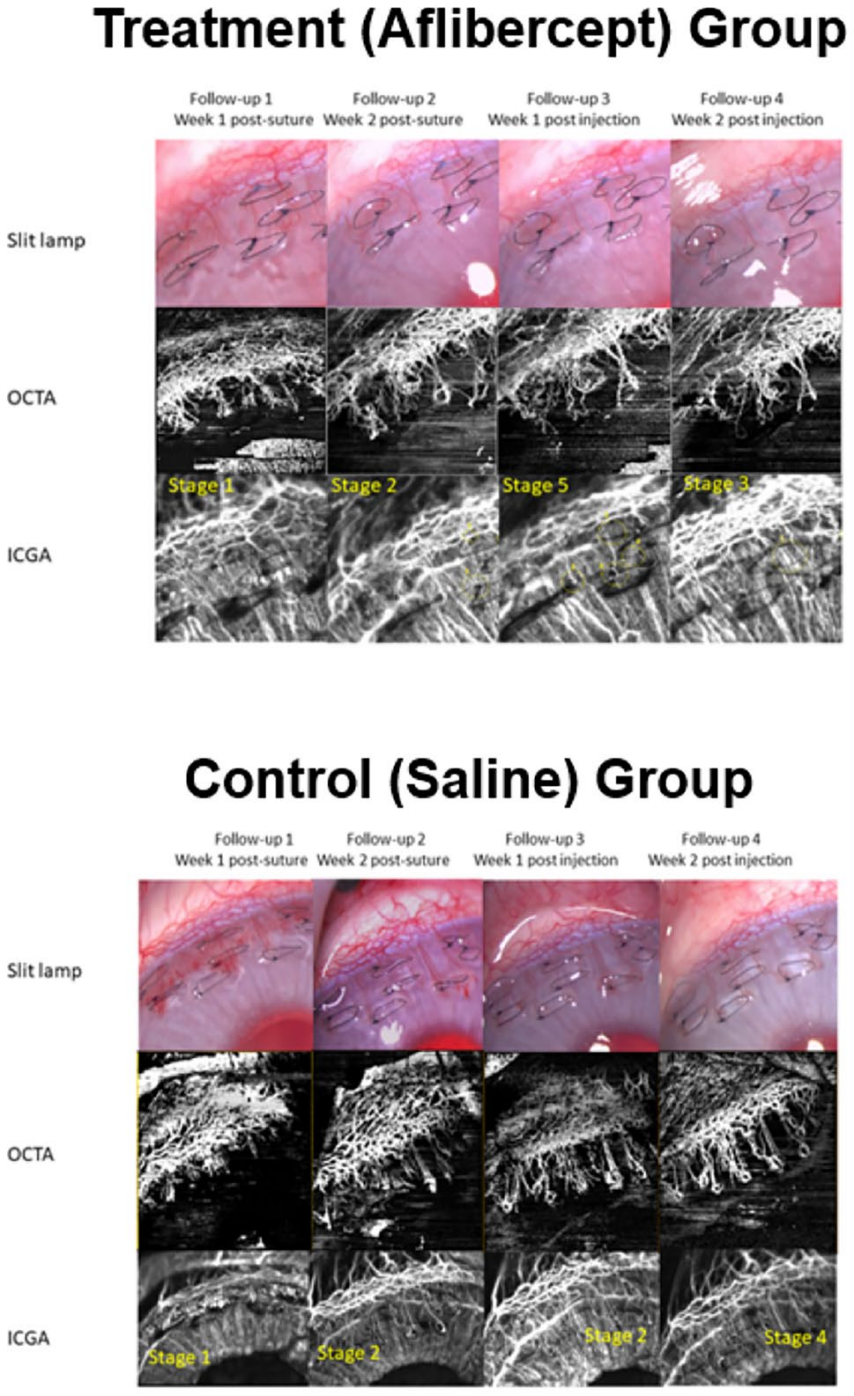
Corneal vascularisation in rabbit eyes before and after receiving subconjunctival aflibercept injections (treatment group) or saline injections (control group). Representative in-vivo slit-lamp photography images (top panels) with their corresponding anterior segment optical coherence tomography angiography (AS-OCTA) images (middle panels) and indocyanine green angiography images (bottom panels).

**Figure 4:**
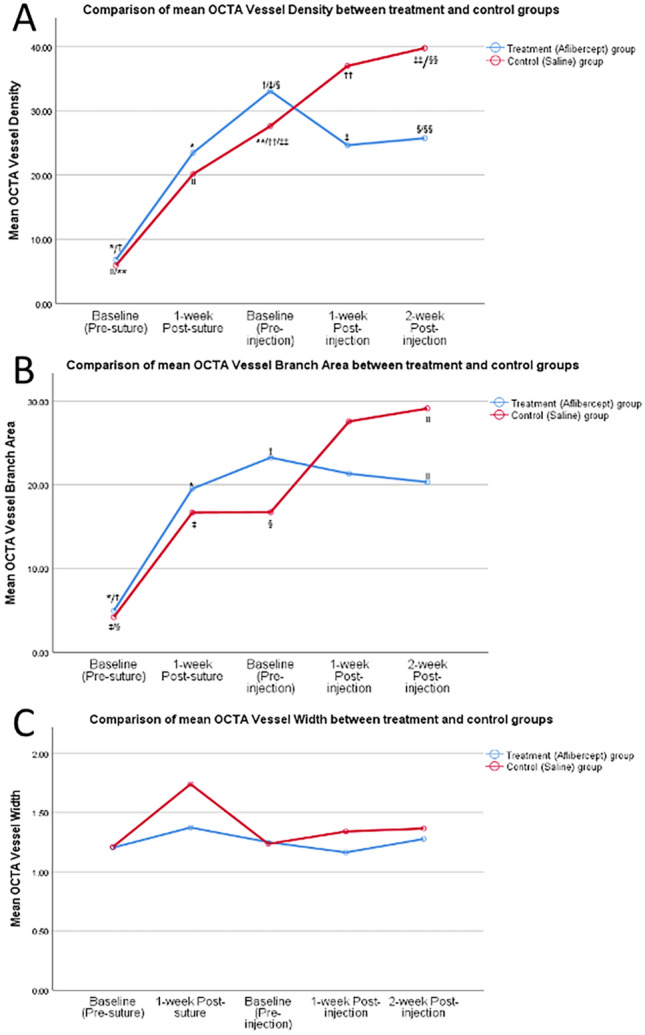
Changes in anterior segment optical coherence tomography angiography (AS-OCTA) determined vascular parameters following corneal suturing to induce corneal vascularisation and subconjunctival aflibercept injections (treatment group) or saline injections (control group). (**A**) Changes in mean vessel densities; (**B**) changes in mean vascular branch area; (**C**) changes in mean vessel width. ^*^, ^†^, ^‡^, ^§^, ^II^, ^**^, ^††^, ^‡‡^, ^§§^ denote statistical significance between two comparative points, p < 0.05).

We analysed all 10 eyes with baseline and serial OCTA with ICGA imaging, which included 5 eyes that underwent re-suture. AS-OCTA vessel densities were significantly higher in the first and second weeks following placement of cornea sutures (30.9 ± 3.4% and 22.2 ± 2.4% respectively), compared to the baseline visit (6.5 ± 2.5%) (p < 0.01). Eyes in the treatment (aflibercept) group exhibited regression in corneal vascularization as demonstrated by a reduction in AS-OCTA vessel densities at one week (24.6 ± 3.4%, p = 0.008) and two weeks (25.7 ± 2.47%, p = 0.003) following sub-conjunctival aflibercept injections, compared to pre-injection measurements (30.9 ± 3.4%) (Figs. 3, 4A). In contrast, eyes in the control (saline) group had increased corneal vascularisation with higher AS-OCTA vessel densities at one week (36.7 ± 2.9%, p = 0.026) and two weeks (39.8 ± 3.3%, p = 0.014) following subconjunctival saline injections, compared to pre-injection measurements (27.6 ± 1.8%). At two weeks following injections, AS-OCTA vessel density of eyes in the treatment group were significantly lower (25.7 ± 2.5%) compared to vessel densities of eyes in the control group (39.8 ± 3.3%) (p = 0.017) (Fig. 4A). We also observed significant differences in vessel branch areas comparing treatment group (20.3 ± 3.1%) and control group (29.1 ± 0.1%) (p = 0.038), but did not find any differences in mean vessel width between the treatment group (1.3 ± 0.1) and control group (1.4 ± 0.1) (p = 0.54) (Fig. 4B,C).

### Correlating AS-OCTA vascular parameters to ICGA vascularisation staging

At the end of the follow-up period, ICGA staging of corneal vascularisation was performed and correlated with 15 OCTA scans obtained through serial imaging. To evaluate the correlation between AS-OCTA vascular parameters with ICGA staging of corneal vascularisation, linear mixed-effects model analysis was performed (Table 1). AS-OCTA vessel densities and AS-OCTA vessel branch areas significantly correlated with the ICGA staging of corneal vascularisation (p ≤ 0.05). This correlation was not observed in AS-OCTA determined vessel widths, where corresponding AS-OCTA vessel widths in eyes with ICGA-graded stages 3, 4 and 5 vascularisation staging were not significantly different from stages 1 and 2 (p = 0.99).

**Table 1:** Linear mixed-effects model analyses comparing serial anterior segment optical coherence tomography angiography (AS-OCTA) determined parameters (vessel density, vessel branch area, vessel width) to indocyanine green angiography (ICGA) determined corneal vascularisation staging.

ICGA corneal vascularisation staging							
Total (n = 15)^a^	Stage 1 and 2 (n = 7)^a^	Stage 3 (n = 3)^a^			Stage 4 and 5 (n = 5)^a^		
AS-OCTA vascular parameters	Mean ± SD	Mean ± SD	Mean difference	P-value	Mean ± SD	Mean difference	P-value
Vessel density	40.46 ± 2.12	13.14 ± 3.26	27.32 ± 4.78	0.005	27.29 ± 1.80	13.17 ± 3.13	0.006
Vessel branching	30.23 ± 2.54	8.73 ± 3.74	21.50 ± 5.32	0.007	20.76 ± 2.08	9.47 ± 3.26	0.05
Vessel width	1.26 ± 0.13	1.44 ± 0.20	− 0.19 ± 0.30	0.99	1.47 ± 0.14	− 0.20 ± 0.24	0.99

^a^Observed number of images at various stages of corneal vascularisation through serial AS-OCTA and ICGA imagings.

### Correlating AS-OCTA vascular parameters to ICGA vessel leakage time

**Figure 5:**
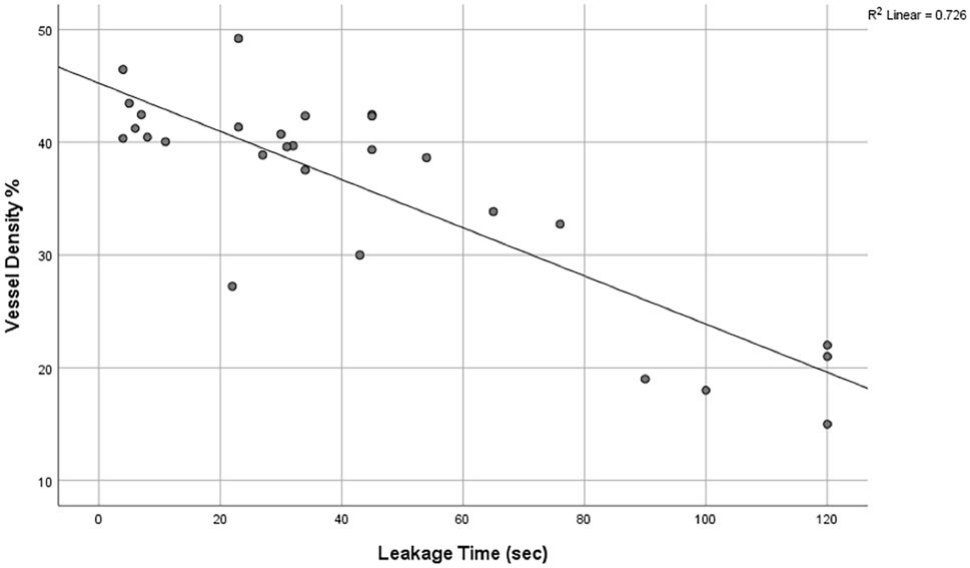
Scatter plot of anterior segment optical coherence tomography angiography determined vessel density values against indocyanine green angiography determined vascular leakage time (in seconds).

Linear regression analyses showed that AS-OCTA determined vessel density significantly predicted ICGA vessel leakage time (model of fit, r^2^ = − 0.726, p < 0.01) (Fig. 5). AS-OCT determined vessel branch area was not a significant predictor of ICGA determined vessel leakage time (p = 0.06).

### Correlating AS-OCTA vascular parameters to pro-angiogenic biomarkers

The correlations between aqueous pro-angiogenic cytokine levels and the respective AS-OCTA vascular parameters were evaluated (Table 2). Aqueous levels of VEGF-A, CRP, and interleukins were below the limits of detection. Aqueous levels of CXCL-12 and PIGF significantly correlated with AS-OCTA vessel densities (r = 0.736 and r = 0.731 respectively, p < 0.05). Vessel branch areas did not show any significant correlation with cytokine levels.

**Table 2:** Spearman’s (r) correlation matrix of changes in aqueous pro-angiogenic biomarkers in relation to anterior segment optical coherence tomography angiography (AS-OCTA) vessel densities and vessel branch area.

Pro-angiogenic cytokines	AS-OCTA Vessel Branch Area, r	AS-OCTA Vessel density, r
ANG	0.431	0.623
CXCL-12	0.565	0.736*
CXCL-13	0.029	0.143
PIGF	0.275	0.731*
VEGF-A	0.252	0.419
IL-8	− 0.110	− 0.008
PDGF-BB	0.581	0.415

*p < 0.05 level (2-tailed).

## Discussion

In this study, we performed serial AS-OCTA imaging in an established animal model of corneal vascularisation in various stages of vessel maturity and correlated various parameters with ICGA staging of vessel severity and activity. We found that AS-OCTA derived vessel densities and vessel branch area measurements significantly correlated with ICGA staging of corneal vascularisation. Linear regression analyses also showed that AS-OCTA vessel densities significantly correlated with ICGA vessel leakage time and changes in pro-angiogenic biomarkers such as CXCL-12 and PIGF, suggesting that AS-OCTA may be a useful imaging technique to detect active corneal vascularisation. These findings further strengthen the potential role of AS-OCTA in studying corneal vascularisation, adding to previous studies that showed that AS-OCTA was able accurately quantify new and regressed corneal vessels, with good inter- and intra-observer agreement in animal models^10,17^.

Dye-based angiographies have been used as the standard investigative technique for assessing the activity of pathological vascularisation due to their ability to detect vessel leakage. Leakage of indocyanine green or fluorescein, have been suggested to be a measure of vascular maturity and disease activity^22–24^. The presence of immature new vessels is associated with shorter dye leakage time, indicating leaky vessels. However, these are invasive techniques with well-documented risks of adverse reactions. The severity of these reactions from intravenous indocyanine green or fluorescein injections can range from mild to potentially life-threatening (e.g. anaphylactic shock, seizures)^8,25,26^. Moreover, these dyes are contraindicated in patients with certain diseases (e.g. renal impairment, liver failure), thus, excluding them from these investigations. Due to these limitations, dye-based angiography imaging are rarely performed to assess corneal vascularisation in current clinical practice^27^.

Previous clinical studies have already demonstrated that OCTA is able to objectively quantify corneal vascularisation comparable to ICGA imaging^9,10,27^. In addition to being non-invasive, one of the advantages of OCTA is the lack of dye leakage, which may obscure underlying vessels or structures in ICGA scans^27^. In animal models, AS-OCTA imaging has also been found to better delineate smaller calibre corneal vessels compared to ICGA^10^. These observations may be related to the larger size of the indocyanine green molecules, which are not able to penetrate through small diameter vessels, and incomplete pericyte coverage may result in reduced visibility of regressed vessels in ICGA imaging^10,19^. Thus, our preliminary study in an animal model provides promising evidence that suggests that non-invasive AS-OCTA imaging may be used to identify active corneal vascularisation, which needs to be further confirmed with a clinical study.

In this study, we also evaluated if AS-OCTA derived vascular parameters, correlated with changes in pro-angiogenic signalling proteins at a molecular level. The significant pro-angiogenic cytokines that were found to be associated with AS-OCTA vessel densities were CXCL-12 and PIGF. Levels of VEGF-A, CRP, interleukins and other growth factors could not be detected as they were below the minimum quantifiable level range; this may have been due the limitation of the protein analysis kit that was used^28^. PIGF is a vascular endothelial growth factor that is known to regulate vascular and lymphatic endothelium differentiation^29^. Its expression is known to result in an increase in branching and size of blood vessels^29^. The specific role of PIGF in pathological angiogenesis has also been reported in previous studies, acting as an inflammatory switch associated with neo-angiogenesis^30^. In our analyses, we found that higher measurements of vessel densities directly correlated with increased expression of PIGF. This therefore suggests that increase PIGF expression is found in cornea angiogenesis, although whether this change in PIGF level is a cause or consequence of angiogenesis is uncertain.

CXCL12 is known to be essential for multiple biological processes, including cellular development, hematopoiesis, organogenesis, and vascularization^31,32^. The correlation of CXCL12 expression with vessel density measurements in our study suggests that CXCL12 expression does promote corneal vascularisation. This corresponds to previous reports of CXCL12/CXCR4 being crucial to the induction of inflammatory corneal angiogenesis^31^. Previous studies of CXCL12 being detected at low levels in normal eyes, but significantly increased in high vessel density and decreased with low vessel density following anti-VEGF treatment, also indicates that CXCL12 pathway is dependent on the VEGF-A/VEGFR-1 pathway in regulating vascularisation^32^. Hence, our results are consistent with previous studies on the role of CXCL12 in angiogenesis and provides a predictive marker of CXCL12 levels through AS-OCTA vessel density quantification.

The findings in our study thus suggest that serial AS-OCTA can be used as a rapid non-invasive tool in clinical practice to determine the severity of corneal vascularisation and monitor disease progression following therapies. For example, AS-OCTA imaging can be used in the field of corneal transplantations. It is well-known that active corneal vascularisation can compromise the corneal immunological privilege and is a major risk factor for corneal graft rejection and graft failure^33,34^. Thus, AS-OCTA determined severity and activity of corneal vascularisation, may be used as a means of stratifying such risks prior to transplant surgeries. Active blood vessels may also be treated prior to transplant procedures^35^. In research settings, AS-OCTA can also be used as an objective assessment tool in clinical trials investigating putative therapies for corneal vascularisation. Indeed, computer-assisted automated image analyses from AS-OCTA may be incorporated into future clinical software designed to quantify disease severity and prognosis^16^.

This study has a number of limitations. Firstly, the association of vascular parameters with corneal vascularisation staging and vessel leakage has only been investigated in our rabbit model using suture-induced corneal vascularisation. The level and characteristics of angiogenesis (e.g. degree of vascular branching) may differ with varying underlying mechanisms of corneal neovascularization, the types of OCTA devices used, and the animal species investigated^36^. The vascular response to various therapeutic agents may also be different. Secondly, our study had a small sample size. Nevertheless, through serial imaging, there was sufficient power to detect significant associations when AS-OCTA determined parameters were compared to ICGA determined vascular staging, ICGA leakage timings, and changes in aqueous cytokine levels. Lastly, current staging of corneal neovascularization has been based only on the brightness and appearance of vessels observed on ICGA. A more thorough understanding of changes of vessel architecture, arborization patterns, and the capillary bed of corneal neovascularization may need to be defined to better characterise the stages of the severity of angiogenesis in the future^37^. Further studies using larger sample sizes and different models of corneal vascularisation are required to validate the findings of this study.

In conclusion, we have demonstrated that AS-OCTA determined vascular parameters may be used to determine the severity and activity of corneal vascularisation in an animal model. This requires confirmation with further studies comparing OCTA vessel parameters and ICGA leakage in a clinical study. Nonetheless, our study provides promising insights into the potential role of AS-OCTA in assessing corneal vascularisation in the future.

## References

[1] Ang M (2018). Anterior segment optical coherence tomography. Prog. Retin. Eye Res..

[2] Spaide RF, Fujimoto JG, Waheed NK, Sadda SR, Staurenghi G (2018). Optical coherence tomography angiography. Prog. Retin. Eye Res..

[3] Tan ACS (2018). An overview of the clinical applications of optical coherence tomography angiography. Eye (Lond.).

[4] Ang M (2016). En face optical coherence tomography angiography for corneal neovascularisation. Br. J. Ophthalmol..

[5] Stanzel TP (2018). Comparison of optical coherence tomography angiography to indocyanine green angiography and slit lamp photography for corneal vascularization in an animal model. Sci. Rep..

[6] Kirwan RP (2012). Quantifying changes in corneal neovascularization using fluorescein and indocyanine green angiography. Am. J. Ophthalmol..

[7] Ang M, Cai Y, Tan AC (2016). Swept source optical coherence tomography angiography for contact lens-related corneal vascularization. J. Ophthalmol..

[8] Olsen TW, Lim JI, Capone A, Myles RA, Gilman JP (1996). Anaphylactic shock following indocyanine green angiography. Arch. Ophthalmol..

[9] Lee WD (2019). Optical coherence tomography angiography for the anterior segment. Eye Vis. (Lond).

[10] Devarajan K (2019). Vessel density and En-face segmentation of optical coherence tomography angiography to analyse corneal vascularisation in an animal model. Eye Vis. (Lond).

[11] Liu YC, Devarajan K, Tan TE, Ang M, Mehta JS (2019). Optical coherence tomography angiography for evaluation of reperfusion after pterygium surgery. Am. J. Ophthalmol..

[12] Hau SC, Devarajan K, Ang M (2019). Anterior segment optical coherence tomography angiography and optical coherence tomography in the evaluation of episcleritis and scleritis. Ocul. Immunol. Inflamm..

[13] Ang M (2015). Optical coherence tomography angiography for anterior segment vasculature imaging. Ophthalmology.

[14] Tey KY (2019). Optical coherence tomography angiography in diabetic retinopathy: A review of current applications. Eye Vis. (Lond).

[15] Cai Y, Alio Del Barrio JL, Wilkins MR, Ang M (2017). Serial optical coherence tomography angiography for corneal vascularization. Graefes Arch. Clin. Exp. Ophthalmol..

[16] Ang M (2018). Optical coherence tomography angiography: A review of current and future clinical applications. Graefes Arch. Clin. Exp. Ophthalmol..

[17] Devarajan K (2019). Optical coherence tomography angiography imaging to monitor anti-VEGF treatment of corneal vascularization in a rabbit model. Sci. Rep..

[18] Ang M (2018). Comparison of anterior segment optical coherence tomography angiography systems for corneal vascularisation. Br. J. Ophthalmol..

[19] Palme C (2018). Functional staging of corneal neovascularization using fluorescein and indocyanine green angiography. Transl. Vis. Sci. Technol..

[20] Chu Z (2016). Quantitative assessment of the retinal microvasculature using optical coherence tomography angiography. J. Biomed. Opt..

[21] Devarajan K (2019). Optical coherence tomography angiography for the assessment of choroidal vasculature in high myopia. Br. J. Ophthalmol..

[22] Anijeet DR (2012). Imaging and evaluation of corneal vascularization using fluorescein and indocyanine green angiography. Investig. Ophthalmol. Vis. Sci..

[23] Bron AJ, Easty DL (1970). Fluorescein angiography of the globe and anterior segment. Trans. Ophthalmol. Soc. U. K..

[24] Easty DL, Bron AJ (1971). Fluorescein angiography of the anterior segment. Its value in corneal disease. Br. J. Ophthalmol..

[25] Balbino M, Silva G, Correia GC (2012). Anaphylaxis with convulsions following intravenous fluorescein angiography at an outpatient clinic. Einstein (Sao Paulo).

[26] Bearelly S, Rao S, Fekrat S (2009). Anaphylaxis following intravenous fluorescein angiography in a vitreoretinal clinic: Report of 4 cases. Can. J. Ophthalmol..

[27] Ang M (2016). Optical coherence tomography angiography and indocyanine green angiography for corneal vascularisation. Br. J. Ophthalmol..

[28] Tarrant JM (2010). Blood cytokines as biomarkers of in vivo toxicity in preclinical safety assessment: Considerations for their use. Toxicol. Sci..

[29] Odorisio T (2002). Mice overexpressing placenta growth factor exhibit increased vascularization and vessel permeability. J. Cell Sci..

[30] De Falco S (2012). The discovery of placenta growth factor and its biological activity. Exp. Mol. Med..

[31] Du LL, Liu P (2016). CXCL12/CXCR4 axis regulates neovascularization and lymphangiogenesis in sutured corneas in mice. Mol. Med. Rep..

[32] Liang Z (2007). CXCR4/CXCL12 axis promotes VEGF-mediated tumor angiogenesis through Akt signaling pathway. Biochem. Biophys. Res. Commun..

[33] Koay PY, Lee WH, Figueiredo FC (2005). Opinions on risk factors and management of corneal graft rejection in the United kingdom. Cornea.

[34] Chong EM, Dana MR (2008). Graft failure IV. Immunologic mechanisms of corneal transplant rejection. Int. Ophthalmol..

[35] Faraj LA, Elalfy MS, Said DG, Dua HS (2014). Fine needle diathermy occlusion of corneal vessels. Br. J. Ophthalmol..

[36] Azar DT (2006). Corneal angiogenic privilege: Angiogenic and antiangiogenic factors in corneal avascularity, vasculogenesis, and wound healing (an American Ophthalmological Society thesis). Trans. Am. Ophthalmol. Soc..

[37] Chua J (2019). Future clinical applicability of optical coherence tomography angiography. Clin. Exp. Optom..

